# Comparative efficacy of the Leucofeligen™ FeLV/RCP and Purevax™ RCP FeLV vaccines against infection with circulating feline Calicivirus

**DOI:** 10.1186/s12917-017-1217-y

**Published:** 2017-10-10

**Authors:** T. Almeras, P. Schreiber, S. Fournel, V. Martin, C. S. Nicolas, C. Fontaine, C. Lesbros, S. Gueguen

**Affiliations:** Virbac, 13ème rue, LID, 06511 Carros, France

**Keywords:** Calicivirus, Coryza, Cat, Vaccine, Efficacy, Live, Leucofeligen, Feligen, Purevax

## Abstract

**Background:**

Feline calicivirus (FCV) is a common virus, found worldwide, mainly responsible for chronic ulceroproliferative faucitis and periodontitis. This virus has a high mutation rate, leading to the presence of numerous FCV strains in the field. The objectives of this study was to evaluate and compare the efficacy of two vaccines (Leucofeligen™ FeLV/RCP and Purevax™ RCP FeLV), which differ by their nature (live vs. inactivated) and the vaccinal strains, against circulating FCV strains. Thirty 9-week-old specific pathogen free (SPF) kittens were thus randomised into 3 groups and were either not vaccinated (control) or vaccinated (2 injections, 3 weeks apart) with one of the vaccines. Four weeks after the second injection of primary vaccination, the cats were inoculated with a pathogenic strain representative of the ones circulating in Europe (FCV-FR4_01) and followed for 2 weeks.

**Results:**

After challenge, significant differences (*p* < 0.05) between control cats and cats vaccinated with Leucofeligen™ FeLV/RCP or Purevax™ RCP FeLV were observed for body weight variation, rectal temperature rise and maximum clinical scores, reflecting the intensity of the signs (83% and 67% lower in the respective vaccinated groups than in the control group). Significant differences were observed between the vaccinated groups, as cats vaccinated with Leucofeligen™ FeLV/RCP had a lower temperature rise (*p* < 0.05 at days post-challenge 3 to 5) and lower virus shedding titres (p < 0.05 at days post-challenge 8, 9 and 11) than cats vaccinated with Purevax™ RCP FeLV. Finally, only cats vaccinated with Leucofeligen™ FeLV/RCP had a significantly lower cumulative score, reflecting the intensity and duration of calicivirosis clinical signs, than the control cats (77% lower vs. 62% lower for cats vaccinated with Purevax™ RCP FeLV).

**Conclusions:**

Both vaccines, Leucofeligen™ FeLV/RCP and Purevax™ RCP FeLV, were found to be efficacious in reducing clinical signs induced by FCV-FR4_01, a FCV strain representative of the circulating ones. However, cats vaccinated with Leucofeligen™ FeLV/RCP were able to control the infection more efficiently than those vaccinated with Purevax™ RCP FeLV, as evidenced by the shorter duration of clinical signs and lower viral titre in excretions.

## Background

Feline calicivirus (FCV) is a RNA virus which can cause various symptoms that affect mainly the oral cavity and, occasionally, the upper respiratory tract [1, 2]. It is one of the main pathogens responsible for coryza (cat flu), a very common disease in cats. Symptoms possibly induced by FCV include oral ulcerations, salivation, gingivitis, stomatitis, conjunctivitis, sneezing and nasal discharge. Some atypical FCV can cause lameness or skin ulcerations while some hypervirulent strains can lead to systemic diseases [1, 2].

Feline calicivirus shows a high antigen variability and can evolve quickly [3, 4]. In a recent study conducted in Europe, 46 strains (pairwise genetic distance >20%) were identified from 72 FCV isolates [3]. However, the phylogenetic analysis of partial variable regions of polymerase or capsid sequences showed that the different strains found in the field had a median uncorrected nucleotide distance of around 40% when compared pairwise or when compared with the F9 strain, currently used in some live vaccines [3].

Vaccines against FCV are available and widely used. They contain one or two FCV strains that are either live attenuated or inactivated. Although regulatory agency approved vaccines should trigger protective immunity, their efficacy can differ according to the type of immunity stimulated [5, 6]. Inactivated vaccines are known to elicit the production of neutralising antibodies (NAb) directed against specific extracellular antigens (humoral immunity). Live vaccines, however, mimic the natural infection and stimulate the cellular component of the immune system (cell-mediated immunity) in addition to the humoral response [5, 6]. Since live attenuated viruses can replicate, live vaccines generally induce a quicker, longer lasting and more efficient immune response than inactivated vaccines [6, 7].

The first aim of this study was to confirm that currently commercialised vaccines against FCV are still efficacious against circulating strains. The second aim was to compare the efficacy of two vaccines containing either the live attenuated strain FCV-F9 (Leucofeligen™ FeLV/RCP, Virbac, France) or two inactivated strains of FCV (431 and G1 [8]; Purevax™ RCP FeLV, Merial, France).

## Methods

This controlled, randomised, not blinded, experimental study was carried out under the supervision of the Ethical Committee of Virbac, in accordance with the recommendations issued in the European Pharmacopoeia [9].

### Animals and study protocols

Thirty specific-pathogen free European kittens (9 weeks old; average ± SD body weight of 1.1 ± 0.12 kg, ranging from 0.87 to 1.34 kg) obtained from a commercial supplier (Liberty Research, USA), were randomly assigned to 3 groups: group 1 (vaccinated with Leucofeligen™ FeLV/RCP), group 2 (vaccinated with Purevax™ RCP FeLV) and group 3 (not vaccinated). Cats were acclimatised for 7 days to the animal housing conditions (12 h light/dark cycle, 18 ± 3 °C, 55 ± 10% humidity with free access to water and commercial dried food daily feeding). During the acclimation and post-challenge phases, a commercial humid food was proposed in addition to ease the acclimation and limit the effect of oral-ulcer-related anorexia.

Cats included in groups 1 and 2 were vaccinated twice, 3 weeks apart (day 0 and day 21), by subcutaneous injection according to the recommendations of the manufacturers. Cats in group 3 did not receive any vaccine.

Four weeks after the second injection of primary vaccination, on day 49, equivalent to day post-challenge (DPC) 0, all cats were inoculated with a virulent heterologous strain of calicivirus (FCV-FR4_01) and observed for 14 days. All cats of each group were housed together but the groups were separated to avoid contaminations.

### Vaccines

Leucofeligen™ FeLV/RCP was granted a pan-European marketing authorisation (centralised procedure) in 2009. It is packaged as a freeze-dried fraction containing the live attenuated viruses, that is, FCV (F9 strain), Feline Herpes Virus-1 (FHV-1; F2 strain), and Feline Panleukopenia Virus (FPV; LR72 strain), and a liquid fraction containing the recombinant FeLV-envelope antigen p45 (derived from FeLV gp70) with aluminium hydroxide and QA-21 adjuvants. Purevax™ RCP FeLV is presented as a freeze-dried fraction containing inactivated strains of FCV (431 and G1) and attenuated strains of FHV (F2) and FPV (PLI IV), and a liquid fraction containing a recombinant virus canarypox FeLV (vCP97).

The vaccine vials were stored between +2 °C and +8 °C and were reconstituted immediately prior to use by rehydrating the freeze-dried fraction with the liquid fraction.

### Challenge

The FCV-FR4_01 strain (Genbank accession number MF882991) was isolated during the field epidemiology study performed by Hou et al. [3]. This strain was chosen for the challenge since it was identified as a single isolate (unique sequence) with a high titration from a single cat presenting various clinical signs typical of a calicivirus infection. The strain was amplified through two passages on CRFK cells and controlled in order to ensure the absence of any interfering pathogens. Regulatory compliant reproducible experimental infections were developed (internal data). The same clinical signs, identical to the ones observed in the original cat, were constantly reproduced: a marked weight loss in the first week, hyperthermia, a depression of the general status and apparition of oral and nasal ulcers, typical of FCV infections. Minor nasal and ocular discharge could also be observed. The FCV-FR4_01 challenge strain was therefore considered as representative of the initial FCV-FR4_01 isolate and thus representative also of the FCV circulating strains.

**Fig. 1:**
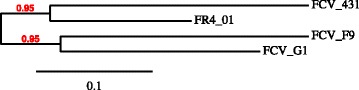
Unrooted tree of the partial capsid sequences of 4 feline calicivirus (FCV) strains, the FCV-FR4_01 challenge strain (GenBank accession No. MF882991) and the 3 vaccine strains FCV-F9 (GenBank accession No. M86379), FCV 431 (National French patent registration number 00 01761), FCV G1 (National French patent registration number 00 01761). This phylogenetic tree was reconstructed using the maximum likelihood method implemented in the PhyML program (v3.1/3.0 aLRT). The percentage of replicate trees in which the associated taxa clustered together in bootstrap tests (1000 replicates) is shown newt to the branches. Only bootstrap values >95 are shown. Distances are drown to scale and relate to the distance bar

Further analyses of the sequence identities, performed on partial capside sequences, showed that the FCV-FR4_01 strain had 73% homology with FCV-F9, 75% with FCV-G1 and 76% with FCV-431 while the three vaccination strains shared between 70% and 74% identities. These levels of homology were in accordance with the homology found between circulating strains [3]. None of the vaccine strains clustered with the FCV-FR4_01 challenge strain (pairwise genetic distance >20%, Fig. 1) [10, 11].

The challenge was performed on anesthetised cats inoculated intranasally with 10^7.5^ cell culture infecting dose, 50% endpoint (CCID_50_) per cat of FCV-FR4_01 suspension using a volume of 0.25 mL/nostril.

### Monitoring

During the vaccination phase, cats were checked daily for appearance of any clinical signs. The animals were examined and weighed weekly. In the post-challenge phase, clinical examinations were performed daily, starting with Group 2 then 1 and ending with Group 3 to avoid contamination between groups, and included rectal temperature measurement and observation of clinical signs (general status, nasal and ocular discharge, ulcers) until DPC 14. Weighing was performed twice a week.

### Scoring

The clinical status was evaluated according to a scoring system based on that specified in the pharmacopoeia monograph [9] (see Table 1). A score was given for each parameter, each cat, on every assessment day. For comparison of scores between groups, two methods were used. The first method, the cumulative score, consisted of adding up all daily scores recorded for each cat and for each parameter (cumulative score per cat for parameter x = sum of scores recorded from DPC 0 to DPC 14). The median of the cumulative scores per parameter was then calculated in each group as well as the median of the total cumulative scores recorded per cat, taking into account all parameters.

**Table 1:** Scoring system for the different signs observed, based on the European Pharmacopoeia monograph 1102 [9]

Observed signs	Description	Score
Death		10
Depressed state		2
Temperature	≥39.5 °C	1
	≤37 °C	2
Ulcer (nasal or oral)	Small and few in number	1
	Large and numerous	3
Nasal discharge	slight	1
	copious	2
Ocular discharge		1
Weight loss ≥3%		2

For the second method, the maximum score recorded per cat and per parameter during the period was taken as a reference to calculate the median maximum scores for each parameter and the total of maximum scores recorded for each cat. The median of the total of maximum scores was used to assess differences in the severity of the clinical picture while the median of the total cumulative scores was used to evaluate the severity and duration of each sign. For both methods, scores for general status, nasal discharge, ocular discharge, ulcers and rectal temperature were included. The score for weight loss (scored once on DPC 7 to evaluate the loss over the first week) was also taken into account for the calculation of the maximum scores.

### Efficacy assessment

A vaccine was judged to be efficacious if the median of the total maximum scores was significantly lower in the vaccinated group than in the control group.

### Viral shedding

Viral shedding was assessed from nasal washings performed daily from DPC 2. Successive dilutions (10-fold steps) of samples were mixed with plated CRFK cells and incubated for one hour at 37 °C before adding more medium. They were then further incubated for 6 days and the cytopathogenic effect of FCV viruses was assessed microscopically. Cats were considered negative when the titre was <10^0.3^ DICC_50_/mL, which was the detection threshold.

### Serological assessments

The serological assessments included evaluation of the titres of total immunoglobulin G (IgG) and of neutralising antibodies (NAb) directed against the FCV-FR4_01 strain. Blood samples were collected on days 0, 21, 35, 49 (DPC 0), 56 (DPC 7) and 63 (DPC 14).

### Total IgG

Titres of IgG reactive to FCV antigens were assessed using an immunofluorescent antibody assay. Briefly, two-fold dilutions of each serum (from 1/8 to 1/8192) were added to a 96-well plate containing acetone-fixed CRFK cells infected with FCV-FR4_01. Positive and negative sera were diluted the same way and used as controls. They were incubated for 1 h at 37 °C and revealed with a fluorescein-conjugated anti-feline IgG antibody and a solution of Evans Blue. The positivity threshold was 1/128 (equivalent to a dilution of 10^2.1^).

### Neutralising Ab

Titres of NAb were determined by serum neutralisation (SN) of the FCV-FR4_01 strain. Briefly, each serum was diluted to provide six 2-fold dilution steps (1/8 to 1/256). Diluted sera were incubated for 1 h with FCV-FR4_01 suspension (100–502 DICC_50_/test) to allow viral neutralisation. Each mixture was then added to six 96-well plates containing 70% confluent CRFK cells. After 6 days of incubation, the characteristic cytopathic effect was assessed. The titre was determined by the Spearman and Karber method [12] and considered negative when inferior to 10^0.9^ which was the detection threshold.

### Statistical tests

All statistical analyses were performed using SAS 9.3 software. For the comparison of parameters with repeated measures (antibody titration, rectal temperature, body weight, and viral shedding), a mixed model analysis of variance was used to compare the 3 groups over time. For weight and temperature, the baseline values were used as covariates. In case of significant interaction, adjusted group comparisons (Tukey-Kramer) at each significant time point were performed. Assumptions of normality and homogeneity of variances of residuals were checked. If the Levene’s test of homogeneity of variances was significant, an ANOVA model allowing for different variances in each group using Kenward & Roger method was used to compute the denominator degree of freedom for the tests involving fixed effects.

For other parameters (scores, days of hyperthermia, area under the hyperthermia curve and weight change between DPC 0 and DPC 7), a Kruskal-Wallis test was performed to compare groups, followed in case of a significant *p*-value by a Dunn’s test for multiple comparisons. The area under the hyperthermia (≥39.5 °C) curve was calculated using the trapezoidal rule (using NCSS 2004 software).

A *p* value <0.05 was considered significant.

## Results

### Vaccination phase (day 0 – day 49)

#### Clinical monitoring and safety

During the vaccination phase, one control cat was found dead while all other cats remained healthy. Weight gain was similar between groups and rectal temperature remained stable in all groups (mean temperatures between 38.5 °C and 39 °C in all groups). No abnormal general or local reaction was observed with any vaccine.

#### Immune response

**Fig. 2:**
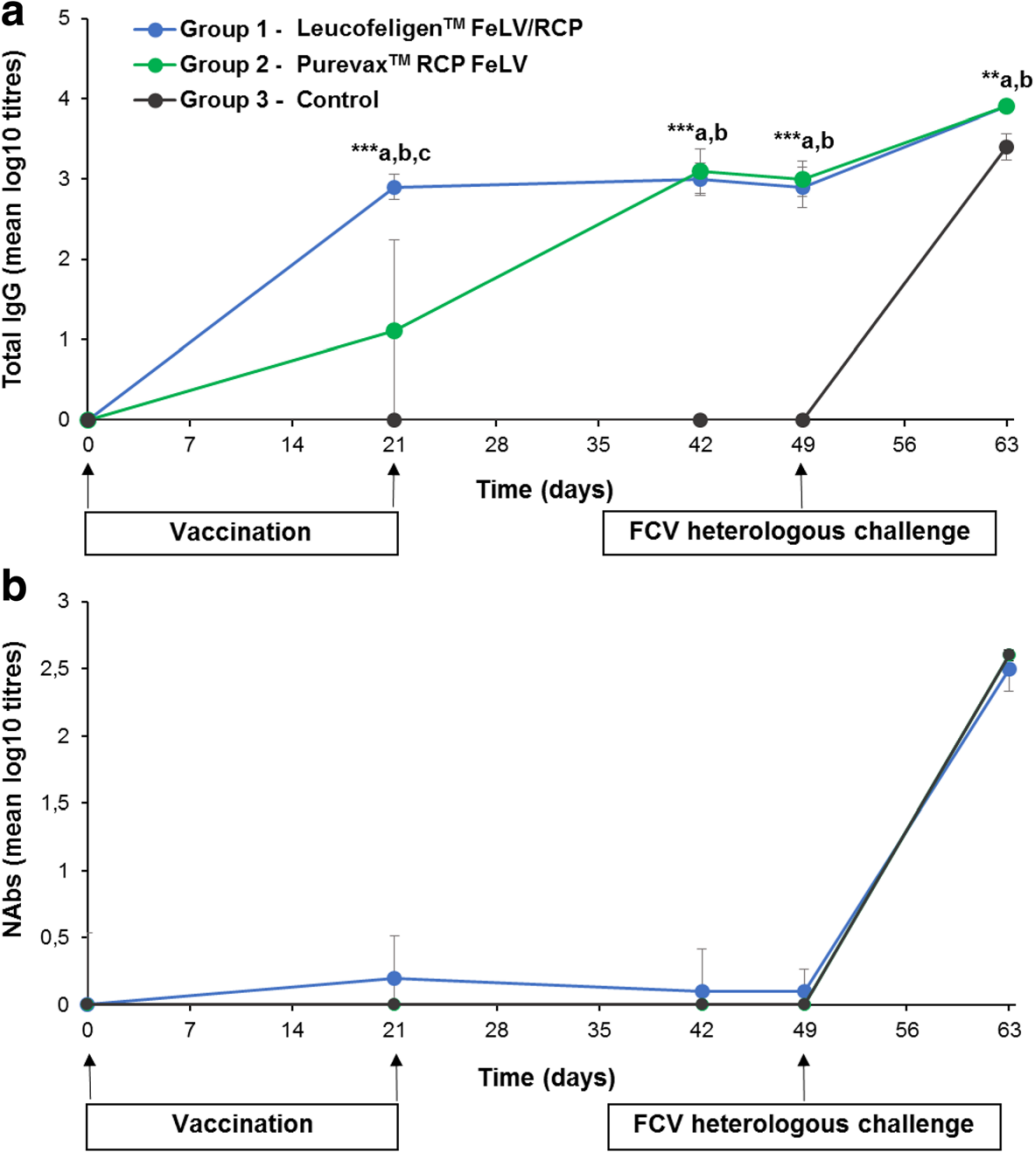
Titration of total IgG and neutralising antibodies directed against the FCV-FR4_01 strain, during the course of the study. Titration of total IgG was assessed by immunofluorescence (IF; **a**) while neutralising antibodies were assessed by a SN assay (**b**). Titrations were performed from samples collected on days 0, 21, 42, 49 (DCP 0) and 63 (DCP 14) in group 1 (Leucofeligen™ FeLV/RCP, *blue*, *n* = 10), group 2 (Purevax™ RCP FeLV, *green*, n = 10) and group 3 (control, *black*, *n* = 9). Mean values (and SD as error bars) are reported. **a** A value of 0 was attributed to titration <2.1 log 10 (positive threshold) and 3.9 log 10 was the maximum value which could be measured. In the vaccinated groups, all values from day 21 were significantly different from day 0 and from values in control group (*p* < 0.05, Tukey adjusted multiple comparisons). **a**, **b** and **c**: statistically significant difference between group 1 (Leucofeligen™ FeLV/RCP) and control (a); between group 2 (Purevax™ RCP FeLV) and control (**b**); and between group 1 (Leucofeligen™ FeLV/RCP) and group 2 (Purevax™ RCP FeLV) (**c**). * p < 0.05; ** *p* < 0.01; *** *p* < 0.001. **b** A value of 0 was attributed to titration <0.9 log 10 (positive threshold) and 2.56 log 10 was the maximum value. No difference between groups was observed

The presence of FCV antibodies was assessed using immunofluorescence (IF) or SN against the FCV-FR4_01 challenge strain. Titration of total FCV antibodies (IgGs) assessed by IF showed a detectable seroconversion from day 21 in 10/10 (100%) cats in group 1 (Leucofeligen™ FeLV/RCP) but only in 5/10 (50%) cats in group 2 (Purevax™ RCP FeLV). Mean FCV-FR4_01 IgGs titre was significantly higher in group 1 (Leucofeligen™ FeLV/RCP) than in group 2 (Purevax™ RCP FeLV) 3 weeks after the first injection of primary vaccination: mean log10 (± SD) of 2.9 ± 0.2 vs. 1.1 ± 1.1 (*p* < 0.001, *n* = 10 in each group, Fig. 2a). Mean titration was similar in both vaccinated groups 3 weeks after the second injection of primary vaccination. All vaccinated cats (20/20) had seroconverted at that time. No antibodies were found in the serum of cats in the control group before inoculating cats with the FCV-FR4_01 strain (*n* = 10). During the vaccination phase, neutralising antibodies were not present in any group or at a very low level on days 21 and 42 (Fig. 2b).

### Post-challenge phase (day 49 – day 63 or DPC 0 - DPC 14)

#### Clinical monitoring

##### Weight

**Fig. 3:**
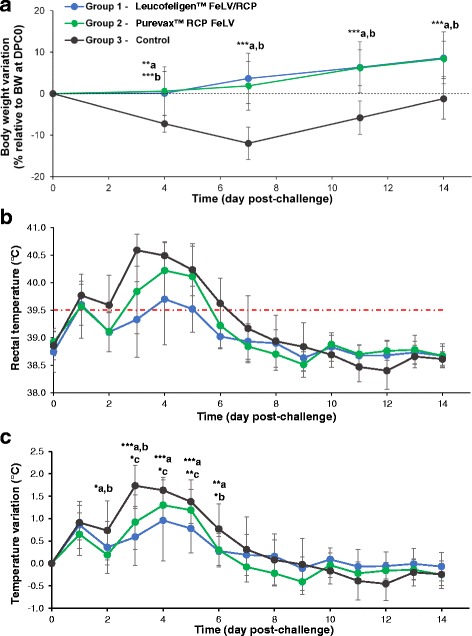
Variations in weight and rectal temperature after challenging cats with the FCV-FR4_01 strain (DPC 0). Weight was assessed on DCP 0, 4, 7, 11 and 14 in group 1 (Leucofeligen™ FeLV/RCP, *blue*, *n* = 10), group 2 (Purevax™ RCP FeLV, *green*, n = 10) and group 3 (control, *black*, *n* = 9) and mean body weight variation (in % from initial body weight at DPC 0) are reported in **a**. Rectal temperature was assessed daily and mean temperatures are reported in **b**. Mean temperature variations (difference from value at DPC 0) are reported in **c**. All data are presented as mean and SD (error bars). Statistically significant differences in variation between groups were observed and are annotated as in Fig. 1

Before challenge on DPC 0, mean (± SD) body weights were not significantly different between groups: 2.11 ± 0.20 kg in the control group; 1.98 ± 0.25 kg in cats vaccinated with Purevax™ RCP FeLV and 1.92 ± 0.29 kg in cats vaccinated with Leucofeligen™ FeLV/RC. A decrease in mean body weight was observed during the first week of the challenge phase in the control group but not in the vaccinated groups (Fig. 3a). Indeed, all kittens (9/9) in the control group lost more than 3% of their body weight (a loss considered significant at this age and taken as a reference for severity [13]) between DPC 0 and DPC 7 compared to only 2 cats in each vaccinated group. Mean weight (± SD) in the control group went from 2.11 ± 0.20 kg to 1.96 ± 0.19 kg and 1.86 ± 0.21 kg from DPC 0 to DPC 4 (*p* < 0.001) and DPC 7 (p < 0.001), respectively, and was of 2.09 ± 0.24 kg at DPC 14 (NS).

##### Temperature

An increase in rectal temperature was observed in all groups with a peak of hyperthermia (≥39.5 °C) on days 3 to 5 after challenge (Fig. 3b). However, compared to DPC 0, cats in the control group had, on average, a higher increase in temperature (*p* < 0.05) than cats in the vaccinated group 1 (Leucofeligen™ FeLV/RCP) on DPC 2 to 6, and than cats in the vaccinated group 2 (Purevax™ RCP FeLV) on DPC 2, 3 and 6 (Fig. 3c). A greater mean increase in rectal temperature was also observed in cats vaccinated with Purevax™ RCP FeLV than in cats vaccinated with Leucofeligen™ FeLV/RCP, on DPC 3, 4 and 5 (*p* < 0.05, Fig. 3c). The number (%) of cats with severe hyperthermia (>40 °C) was of 5/10 (50%), 8/10 (80%) and 9/9 (100%) in group 1, 2 and 3 (control), respectively. Furthermore, cats in the control group remained, on average ± SD, longer in hyperthermia than those in vaccinated groups 1 and 2 (5.2 ± 1.6 vs. 2.1 ± 1.3 and 3 ± 1.4 days, respectively, *p* < 0.05). Cats vaccinated with Purevax™ RCP FeLV remained longer, on average ± SD, in severe hyperthermia than cats vaccinated with Leucofeligen™ FeLV/RCP (3.2 ± 1.2 vs. 0.8 ± 1.0 days, p < 0.05). Overall, by comparing the areas under the hyperthermia curves (>39.5 °C), the cats in group 1 (Leucofeligen™ FeLV/RCP), unlike those in group 2 (Purevax™ RCP FeLV), responded differently than the cats in the control group (1.10 ± 1.01; 2.26 ± 1.37 and 3.77 ± 1.29 °C.day, mean ± SD respectively, p < 0.05). These data suggest that control cats had a more severe and longer lasting hyperthermia than cats vaccinated with Leucofeligen™ FeLV/RCP.

##### Clinical signs

Concerning general status, 7/9 (78%) cats in the control group showed apathy or depression while the cats in the vaccinated groups 1 and 2 showed no general signs. Rare, minor and transient ocular and nasal discharges were observed in very few cats, in groups 1 (Leucofeligen™ FeLV/RCP, 1 cat with ocular discharge for 1 day) and 3 (control, 1 cat with ocular discharge and 1 with nasal discharge for 1 day each). Fewer cats vaccinated with Leucofeligen™ FeLV/RCP had buccal and nasal ulcers (3/10: 30%) than cats in the other groups (7/10: 70% of cats vaccinated with Purevax™ RCP FeLV and 7/9: 78% of control cats). Large ulcers (≥5 mm) were found in 0/10 (0%), 1/10 (10%) and 2/9 (22%) cats in groups 1, 2 and 3, respectively. Ulcers lasted for up to 3 days in group 1 but up to 10 days in groups 2 and 3.

### Scores

**Fig. 4:**
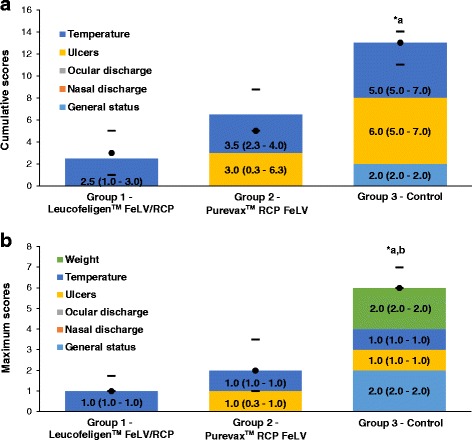
Clinical scores in the post-challenge phase. **a**: Median (IQR in brackets) of cumulative clinical scores for temperature, ulcers, ocular discharge, nasal discharge and general status, in each group. The *black circle* indicates the median of total cumulative scores recorded in each cat and the dashes the 1nd and 3rd quartiles. **b**: Median (IQR in brackets) of maximum scores observed for each sign. The 6 parameters assessed are represented (weight, temperature, ulcers, ocular discharge, nasal discharge and general status). The *black circle* indicates the median of total maximum scores recorded in each cat and the dashes the 1nd and 3rd quartiles. Statistically significant differences between groups were observed on the median of the total scores per cat and are annotated as in Fig. 1. Leucofeligen™ FeLV/RCP: n = 10; Purevax™ RCP FeLV: n = 10 and control: n = 9

Cumulative clinical scores take into account the scores recorded daily for each parameter (general status, nasal discharge, ocular discharge, ulcers and rectal temperature). The median (inter-quartile range or IQR) of the total cumulative clinical scores, indicative of the clinical severity and duration of signs, was significantly higher in the control group [13.0 (11.0–14.0); *n* = 9] than in the group vaccinated with Leucofeligen™ FeLV/RCP [3.0 (1.0–5.0); *n* = 10; *p* < 0.05] but was not significantly different than in the group vaccinated with Purevax™ RCP FeLV [5.0 (5.0–8.8); *n* = 10, Fig. 4a]. Compared to unvaccinated cats, the median of the total cumulative clinical scores was 77% lower in cats vaccinated with Leucofeligen™ FeLV/RCP and was 62% lower in those vaccinated with Purevax™ RCP FeLV.

The median (IQR) of the total maximum scores (all parameters/cat: general status, nasal discharge, ocular discharge, ulcers, rectal temperature and weight) was significantly higher (p < 0.05) in the control group [6.0 (6.0–7.0); *n* = 9] than in the group vaccinated with Leucofeligen™ FeLV/RCP [1.0 (1.0–1.8); 83% lower; *n* = 10] and the group vaccinated with Purevax™ RCP FeLV [2.0 (1.0–3.5); 67% lower; n = 10; Fig. 4b).

### Viral shedding

**Fig. 5:**
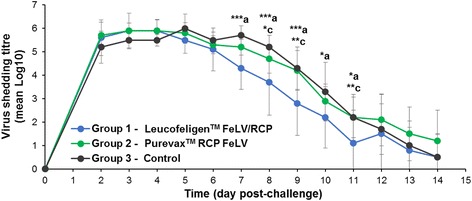
Viral shedding during the post-challenge phase. Viral shedding was assessed from nasal washings performed daily in the different groups (*blue*: group 1 – Leucofeligen™ FeLV/RCP, n = 10; *green*: group 2 – Purevax™ RCP FeLV, n = 10 and *black*: group 3 – control, n = 9). Data are presented as mean and SD (error bars). Statistically significant differences between groups were observed and are annotated as in Fig. 1

A peak of viral shedding was observed in all groups between DPC 2 and DPC 5 (Fig. 5). However, viral elimination seemed to occur earlier and faster in some animals vaccinated with Leucofeligen™ FeLV/RCP than in the control group or the group of cats vaccinated with Purevax™ RCP FeLV. Indeed, viral titres were significantly lower in cats vaccinated with Leucofeligen™ FeLV/RCP than in the control group on DPC 7 to 11 (*p* < 0.05, n = 10 and 9, respectively, Fig. 5) and significantly lower than in group 2 on DCP 8, 9 and 11 (p < 0.05, n = 10 in each group, Fig. 5). No statistical differences were observed between groups 2 (Purevax™ RCP FeLV) and 3 (control).

### Immune response

As shown in Fig. 2, all cats had seroconverted at the end of the study. The titre of total IgG was, however, significantly lower in the control group than in both vaccinated groups (*p* < 0.01, Fig. 2a).

## Discussion

The feline calicivirus has the propensity to evolve quickly, so that different strains can be found in the field [3,4]. Because of the heterogeneity between strains [3], it was of importance to verify that vaccines formulated with strains isolated a few years ago were still efficacious against circulating strains.

In this study, cats were inoculated with one of the strains (FCV-FR4_01) isolated during a field epidemiology study conducted in Europe and published in 2016 [3]. This strain was chosen as it was genetically representative of the circulating classical FCV pathogenic strains. This statement is based on the lack of evidence for FCV sub-species clustering or serotypes except for viruses sharing immediate temporal or spatial links [3]. Moreover, the clinical signs induced by the FCV-FR4_01 challenge strain were also representatives of a classical feline calicivirosis [2]. In all cats (10/10) in the control group, the dose and route of administration used for the experimental infection induced one or several typical signs of feline calicivirosis, ie a marked weight loss in the first week, hyperthermia, a depression of the general status and apparition of oral and nasal ulcers. These signs were similar to those presented by the initial cat [3] and cats previously challenged with this strain (internal data). This percentage of unvaccinated cats showing notable clinical signs of feline calicivirosis (hyperthermia, bucal ulcers and respiratory signs) is higher than the minimum of 80% required by the European pharmacopeia to validate a FCV experimental infection. This result suggests that the experimental FCV-FR4_01 infection performed was particularly severe and that this strain can be an excellent candidate to test FCV vaccines efficacy in the future.

The efficacy evaluations of the Leucofeligen™ FeLV/RCP and of the Purevax™ RCP FeLV vaccines against this heterologous strain were based on the scores obtained for different parameters. Their efficacy against FCV heterologous strains has been reported in the past [13–15] but not against circulating ones. Consistently with the results obtained in these previous studies [13, 15], the median of the total of maximum clinical scores obtained in the vaccinated groups were significantly lower than the one obtained in the control group (83% and 67% lower for the Leucofeligen™ FeLV/RCP and Purevax™ RCP FeLV vaccines, respectively). The maintenance of the protection conferred by the commercial vaccines against circulating FCV strains supports the idea that the observed inter-strain genetic difference [3] may not induce a significant change in the FCV capsid amino acid sequence and/or configuration. These data also show that the clinical cross-protection triggered by a FCV vaccine is independent of the number of strains it contains. Indeed, the Leucofeligen™ FeLV/RCP vaccine, containing one FCV strain (FCV-F9), brings a similar (or even better under the experimental conditions of this study) clinical cross-protection than the Purevax™ RCP FeLV vaccine which contains two FCV stains (FCV-G1 and FCV-431) [8, 15].

Interestingly, vaccinated cats were protected despite the lack of detectable SN during the vaccination phase (Fig. 2). This observation is in agreement with previous studies showing that the absence of seroneutralising FCV antibodies is not correlated with an absence of protection [13, 15] and in terms of antibody protection, the levels of IgA mucosal antibodies, if available, would be a more appropriate marker of the efficacy of the mucosal defense and ultimate shedding [13]. Indeed, despite a certain level of cross-neutralisation, NAbs are quite specific of the strain used to induce them [13, 16, 17]. The protection against heterologous strains is also and sometimes mainly induced by the cell-mediated immune response triggered by the vaccine and the IgGs, not necessarily neutralising, directed against various viral or infected-cell antigens (total IgGs) [5–7, 18]. Therefore, it would be more relevant to analyse the cell-mediated immune response to evaluate a FCV vaccine efficacy rather than evaluating the level of Nabs in vitro [15–17]. However, since no technique can be routinely used to assess*,* in vitro, the cell-mediated immune response, experimental infection in vivo remains the only reliable way to assess FCV vaccines efficacy.

Both vaccines were deemed efficacious. However, some differences were observed between the cats vaccinated with Purevax™ RCP FeLV and those vaccinated with Leucofeligen™ FeLV/RCP. First, the cats vaccinated with Leucofeligen™ FeLV/RCP could seroconvert faster (within the 3 weeks after the first primary injection) than some cats vaccinated with Purevax™ RCP FeLV (two injections of primary vaccination required for seroconversion of all cats) (Fig. 2a). The difference in the types of vaccine used, live versus killed, may provide a partial explanation for this result [5, 6]. For example, live organisms induce a greater up-regulation of activation molecules and granulocyte-macrophage colony-stimulating factors than do killed organisms [19]. This difference may explain both, the delay in producing IgGs and the requirement for two injection of primary vaccination for the cats vaccinated with Purevax™ RCP FeLV [5–7]. On the contrary, the Leucofeligen™ FeLV/RCP vaccine may require only one injection as a primary vaccination since IgGs were detected after the first injection. Further tests are required to confirm this. Similar findings regarding the speed of onset of immunity, number of injections for primary vaccination and clinical efficacy properties of vaccines depending on their nature, live or killed, might also be observed in other mammals including humans.

The severity and duration of symptoms such as temperature rise or ulcerations also differed between groups. The median of the total cumulative scores was lower in cats vaccinated with Leucofeligen™ FeLV/RCP than in cats vaccinated with Purevax™ RCP FeLV. Although this difference was not statistically significant, only the group of cats vaccinated with Leucofeligen™ FeLV/RCP had a significantly lower score than the control group (77% lower vs. 62% lower for the group vaccinated with Purevax™ RCP FeLV). These data suggest that the Leucofeligen™ FeLV/RCP vaccine would bring a better protection against the heterologous FCV-FR4_01 strain than the Purevax™ RCP FeLV vaccine. Indeed, when assessing the viral shedding, it was found that the titres of viruses eliminated by cats vaccinated with Leucofeligen™ FeLV/RCP was lower than in cats vaccinated with Purevax™ RCP FeLV on DPC 8, 9 and 11. No difference was found between this latter group and the control group, though. These results are consistent with previous studies and the general knowledge that live attenuated vaccines provide a quicker and more efficient protection than inactivated vaccines [6, 20, 21]. They further confirm that a vaccine with a combination of several FCV strains (e.g. Purevax™ RCP FeLV) is not more efficient against heterologous strains than a vaccine with one FCV valence (e.g. Leucofeligen™ FeLV/RCP) and does not seem to broaden the protection against antigenically distant strains, as previously mentioned [15].

In this study, an FCV strain representative of the circulating ones was used for the challenge [3] to ensure that currently commercialised FCV vaccines were efficacious against heterologous circulating strains. Due to the comparable level of genetic difference between FCV strains, similar clinical results are expected if another FCV circulating strain is used for the challenge, as shown previously [13, 15]. However, the results may differ if a highly virulent strain is chosen for challenge and further analyses are required to assess the efficacy of vaccines against this type of strains [4].

## Conclusion

In this study, we compared the ability of two vaccines (Leucofeligen™ FeLV/RCP and Purevax™ RCP FeLV) directed against FCV and other viruses to protect against a heterologous FCV strain circulating in the field (FCV-FR4_01). Both vaccines were found to be efficacious in reducing the clinical signs induced by this strain, compared to an unvaccinated group. However, some differences concerning the rapidity of seroconversion, the duration of clinical signs, and the reduction in viral titre in excretions were found, in favor of the Leucofeligen™ FeLV/RCP vaccine. This study therefore showed that both vaccines still have a good efficacy against circulating strains but that Leucofeligen™ FeLV/RCP may bring a better protection than Purevax™ RCP FeLV against a heterologous strain.

## Abbreviations

Ab: Antibodies
CCID_50_: Cell culture infecting dose, 50% endpoint
CRFK: Crandell Rees feline kidney
DPC: Day post-challenge
FCV: Feline calicivirus
FeLV: Feline leukemia virus
FHV: Feline herpesvirus
FPV: Feline panleukopenia virus
IgG: Immunoglobulin G
IQR: Inter-quartile range
NAb: Neutralising antibodies
RCP: Feline rhinotracheitis virus, calicivirus and panleukopenia virus
SD: Standard deviation
